# Has Suicide Really Increased After the COVID-19 Pandemic?

**DOI:** 10.1192/j.eurpsy.2022.57

**Published:** 2022-09-01

**Authors:** D. Wasserman

**Affiliations:** Karolinska Institutet, National Centre For Suicide Research And Prevention Of Mental Lll-health, Solna, Sweden

## Abstract

Despite being preventable, approximately 800,000 people die by suicide each year worldwide. Evidence suggests that suicide rates decrease during crises, but once the immediate threat passes, suicide rates are expected to increase. The COVID-19 pandemic likely affects risk and protective factors for suicide. Studies show mixed results regarding whether suicide behaviours have increased among adults during the pandemic. The results are however different for young people. An increase in suicidal behaviours has been identified in some countries among young people after lockdown period and when returning to schools. Data also suggests that there may have been a rise in deaths by suicide among those younger than 18 years in China, and during the first phase of lockdown in the United Kingdom. Studies have found significant decreases in hospital admissions for suicidal behaviours and visits to emergency departments both for adults and adolescents. Suicide can be prevented if evidence-based methods that exist are implemented in a systematic way. Wasserman, D., Iosue, M., Wuestefeld, A., & Carli, V. (2020). Adaptation of evidence-based suicide prevention strategies during and after the COVID-19 pandemic. World psychiatry: official journal of the World Psychiatric Association (WPA), 19(3), 294–306.

**Disclosure:**

No significant relationships.

